# Oviposition Activity and Ecological Patterns of Medically Important Culicidae in an Agroforestry System in the Atlantic Forest, Rio de Janeiro, Brazil

**DOI:** 10.3390/tropicalmed11080210

**Published:** 2026-07-25

**Authors:** Júlia dos Santos Silva, Cecilia Ferreira de Mello, Shayenne Olsson Freitas Silva, Amanda Queiroz Bastos, Ana Laura Carbajal-de-la-Fuente, Thamires Rezende Araújo, Jeronimo Alencar

**Affiliations:** 1 Fundação Oswaldo Cruz (Fiocruz), Instituto Oswaldo Cruz, Laboratório de Diptera, Avenida Brasil 4365, Manguinhos, Rio de Janeiro 21040-360, Brazil; juliass@ioc.fiocruz.br (J.d.S.S.); ceciliafmello@gmail.com (C.F.d.M.); shayenneolsson@gmail.com (S.O.F.S.); amanda2qu@gmail.com (A.Q.B.); tamirezendearaujo@gmail.com (T.R.A.); 2 Consejo Nacional de Investigaciones Científicas y Técnicas (CONICET), Godoy Cruz 2290, Ciudad Autónoma de Buenos Aires CP 1425, Argentina; analaura.carbajal@gmail.com; 3 Administración Nacional de Laboratorios e Institutos de Salud “Dr. Carlos Malbrán”, Instituto Nacional de Parasitología “Dr. Mario Fatala Chaben” (INP), Ministerio de Salud de la Nación, Av. Paseo Colón 568, Ciudad Autónoma de Buenos Aires CP 1063, Argentina

**Keywords:** mosquito diversity, ovitraps, entomological surveillance, human occupation gradients

## Abstract

Understanding mosquito diversity in natural environments under anthropogenic influence or undergoing vegetation recovery is essential for comprehending potential behavioral changes and adaptations in the activity patterns of these populations. Within this context, this study aimed to investigate the oviposition activity, seasonal abundance, hatching dynamics, and sex ratio of medically important Culicidae species using ovitraps as an entomological surveillance tool, with special attention to *Haemagogus leucocelaenus*, *Aedes terrens*, and *Aedes albopictus*, and to analyze their distribution within an agroforestry area. The study was conducted at Sítio Terra Boa, a rural property managed under an agroforestry system, in the municipality of Silva Jardim, state of Rio de Janeiro, Brazil, between August 2021 and June 2025. Ten ovitraps were installed at a height of approximately 2 m above ground level and randomly distributed around an area cultivated with açaí (*Euterpe oleracea*) and peach palm (*Bactris gasipaes*). Collections were conducted monthly, and the paddles were replaced and subsequently sent to the laboratory for analysis. The 47-month study provided valuable data regarding the ecology of these species in an agroforestry environment. *Haemagogus leucocelaenus* was the dominant species throughout the study, and mosquito abundance peaked during the warmer months, particularly from December to April. The results revealed seasonal fluctuations in populations, species-specific oviposition patterns in response to environmental changes, displacement of sylvatic species such as *Hg. leucocelaenus* toward anthropized areas, and the expansion of urban vectors such as *Ae. albopictus* into forest matrices. The higher abundance of *Hg. leucocelaenus* in the ovitraps suggests that this species may persist in agroforestry areas; however, comparative studies across different landscape types are needed to evaluate its ecological adaptation. The coexistence of *Haemagogus* and *Aedes* species within the same forest fragment under anthropogenic interference reinforces the need for continuous entomological surveillance in these environments. These findings contribute to understanding vector ecology in recovering Atlantic Forest environments and provide support for future control programs.

## 1. Introduction

Environmental changes resulting from anthropogenic activities, such as deforestation, agricultural expansion, and unplanned urbanization, have significantly altered the ecological dynamics of several mosquito species, influencing their distribution, abundance, and behavior [1,2]. These transformations may favor the movement of sylvatic vectors into peri-urban and urban areas, increasing the transmission risk of diseases such as yellow fever, dengue, Zika, Chikungunya, and malaria [3,4]. Climate change, including rising temperatures and modifications in precipitation patterns, may further expand the geographic distribution of these vectors [5,6].

In this context, agroforestry systems emerge as interface environments between forest fragments and anthropized areas, potentially acting either as barriers or facilitators of the dispersal of medically important Culicidae species [1,2]. Studies indicate that the structural complexity of these systems may influence mosquito diversity by reducing the presence of invasive species while creating favorable microhabitats for certain vector species [3].

For example, *Aedes aegypti* (Linnaeus, 1762) is an urban vector highly adapted to anthropized environments, whereas species of the *Haemagogus* and *Sabethes* genera play a crucial role in the sylvatic transmission of yellow fever. Because biodiversity can influence pathogen transmission by altering host availability, vector abundance, and host-vector interactions, understanding mosquito ecology in these transitional environments is essential for predicting changes in disease transmission dynamics [3,4].

The state of Rio de Janeiro is characterized by its biogeographical diversity and intense urban pressure and, therefore, presents favorable conditions for vector mosquitoes to proliferate in both sylvatic areas and transition zones [1]. Species such as *Haemagogus leucocelaenus* (Dyar & Shannon, 1924), a vector found naturally infected with the sylvatic yellow fever virus in the region, in addition to *Aedes terrens* (Walker, 1856) and *Aedes albopictus* (Skuse, 1894), have demonstrated the ability to adapt to different gradients of human occupation, reinforcing the need for continuous monitoring of these populations [2,3].

The use of ovitraps has proven to be an effective tool for collecting mosquito eggs, particularly those of *Ae. albopictus*, *Ae. terrens* and *Hg. leucocelaenus*, when deployed in sylvatic environments [7]. Oviposition is one of the fundamental aspects for understanding the ecology of these vectors, since breeding site selection is directly associated with resource availability and the microclimatic conditions of the environment [4]. Studies evaluating oviposition preference in different vertical strata and agroforestry matrices may provide important insights for predicting possible displacement of sylvatic species toward populated areas, as well as assisting in planning surveillance and control strategies [5,6].

The selection of an agroforestry area as the study site is supported by growing evidence that these agroecological systems contribute to regulating vector insect populations [8]. Compared with monocultures, agroforestry systems provide greater structural heterogeneity, favoring a higher diversity of natural predators and fragmented aquatic habitats suitable for oviposition, which may reduce the proliferation of medically important mosquitoes [9]. In addition, they represent a sustainable alternative to conventional agriculture and may contribute to reducing public health risks by promoting greater biodiversity [10,11]. Furthermore, the presence of intercropped species such as açaí (*Euterpe oleracea*) and peach palm (*Bactris gasipaes*) may create distinct microenvironments that favor the occurrence of species with different ecological requirements [12,13,14]. Additional studies on the interaction between vegetation and mosquito fauna in these systems may contribute to integrated vector management strategies and biodiversity conservation [15].

The aim of this study was to investigate the oviposition activity and ecological patterns of medically important Culicidae mosquitoes in an agroforestry area in the municipality of Silva Jardim, state of Rio de Janeiro, Brazil, using ovitraps as an entomological surveillance tool. Based on the data obtained, an integrated ecological analysis of medically important species (*Hg. leucocelaenus*, *Hg. janthinomys*, *Ae. albopictus*, *Ae. terrens*, and *Ae. aegypti*) was conducted, including characterizing the abundance and seasonality, evaluating hatching dynamics using cumulative hatchability percentages across successive immersions, and determining the sex ratio of emerged populations.

## 2. Materials and Methods

### 2.1. Ethics Statement

A permanent license for the collection, capture, and transportation of zoological material was granted by the Chico Mendes Institute for Biodiversity Conservation (ICMBio) and the Biodiversity Authorization and Information System (SISBIO) under permit No. 84318-4.

### 2.2. Study Area

**Figure 1:**
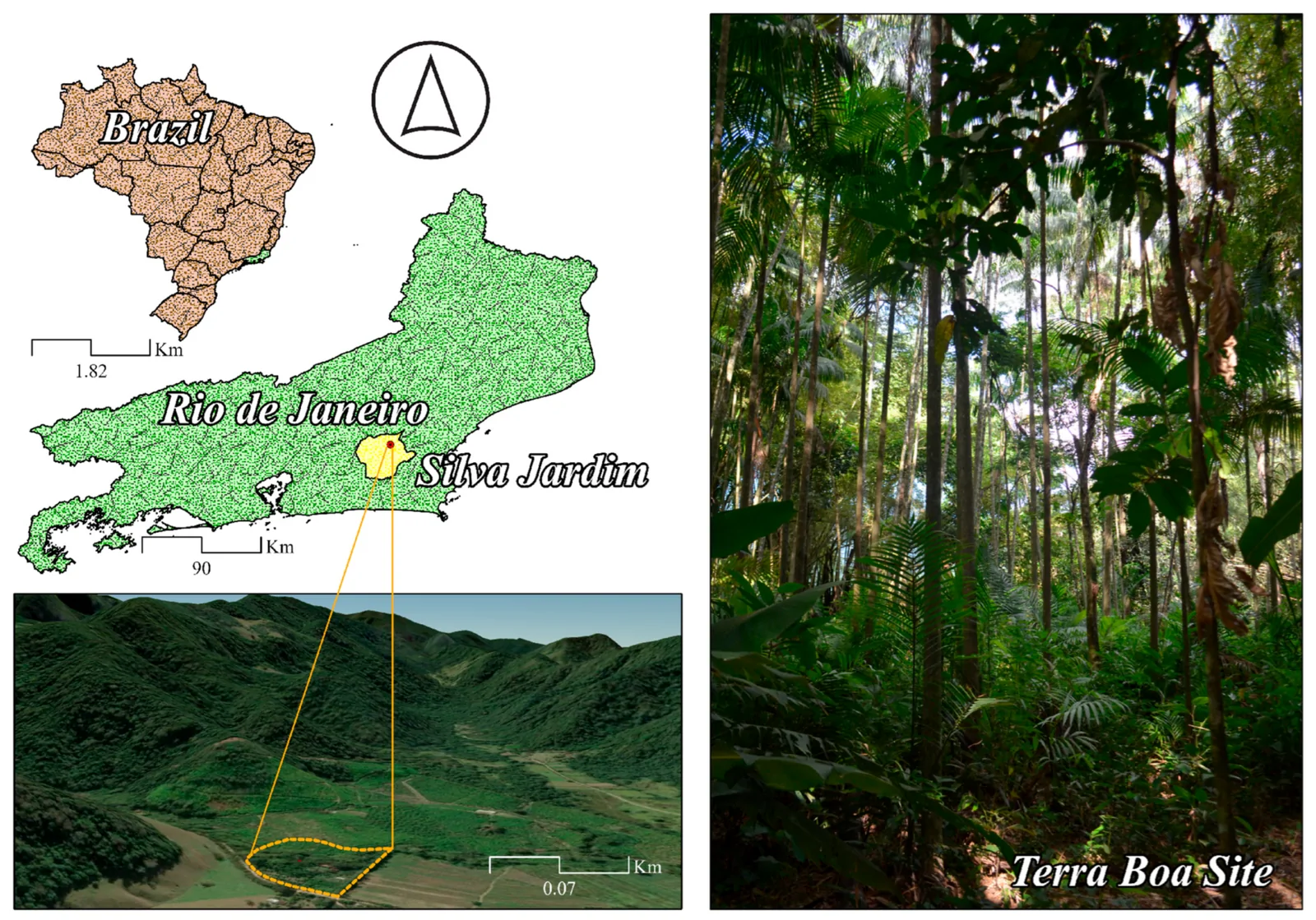
Location and characterization of the study area at the Sítio Terra Boa, a rural property managed under an agroforestry system, municipality of Silva Jardim, state of Rio de Janeiro, Brazil. Map created using software ArcGIS Pro 3.0.1 (ESRI, Redlands, CA, USA), SIRGAS 2000.

The study was conducted in an Atlantic Forest fragment located at the Sítio Terra Boa, municipality of Silva Jardim, state of Rio de Janeiro, Brazil (22°31′40.1″ S; 42°02′58.6″ W) (Figure 1). More specifically, it was conducted in an agroforestry area, an agroecological system that combines the cultivation of agricultural species with native trees, promoting biodiversity, soil restoration, and environmental sustainability. The area consisted of an intercropping system of açaí (*Euterpe oleracea*) and peach palm (*Bactris gasipaes*). These species are commonly used in regional agroforestry systems, which, in addition to generating income, contribute to the conservation of local fauna and flora [16]. According to the Köppen climate classification, the region has an Aw climate (tropical savanna with a dry winter), with a mean annual temperature of approximately 24 °C and annual precipitation ranging from 1200 to 1500 mm, with rainfall concentrated between October and March. The study site is surrounded by a matrix of small rural properties, representing a landscape in which agroforestry areas coexist with remnants of Atlantic Forest vegetation [17].

### 2.3. Collection of Mosquito Eggs Using Ovitraps in an Agroforestry Area

Collections were conducted monthly between August 2021 and June 2025 (a total period of 3 years and 10 months). Mosquito eggs were collected using ovitraps consisting of black plastic containers (500 mL capacity, without lids) resembling plant pots. Natural water (300 mL), leaf litter, and organic matter collected from the forest floor were added to each container in order to simulate an attractive environment for oviposition. An overflow hole was made in each container at the 400 mL level to prevent overflow due to rainfall. Water levels were checked monthly and replenished when necessary to maintain the initial volume. Each ovitrap contained three Eucatex^®^ (São Paulo, Brazil) paddles (dimensions: 2.5 cm × 14 cm) positioned vertically and fixed with clips according to the methodology adapted from Alencar et al. (2022) [7].

### 2.4. Installation and Monitoring of Ovitraps

A total of 10 ovitraps were installed at a height of approximately 2 m above ground level at 10 distinct sampling points, within an area cultivated with açaí (*Euterpe oleracea*) and peach palm (*Bactris gasipaes*). Trap locations were determined using randomly generated spatial coordinates in ArcGIS Pro 3.0.1 within the boundaries of the cultivated area. A spatial constraint was applied to ensure a minimum distance of 10 m between traps. The 10-m spacing between ovitraps was established to minimize spatial autocorrelation among sampling units and to provide representative coverage of the cultivated area following previous ovitrap surveillance studies conducted in Atlantic Forest environments [7]. This distance reduces the probability that a single ovipositing female would visit multiple traps during the same gonotrophic cycle, thereby minimizing pseudoreplication. The randomization was restricted to the cultivated area, excluding border zones and sectors with excessively dense vegetation that could hinder monthly access and trap maintenance. The height and spatial arrangement of the traps were designed to target mosquito species associated with intermediate vegetation strata and vertical oviposition behavior, such as *Hg. leucocelaenus* and *Ae. albopictus* [17].

### 2.5. Laboratory Processing of Samples

The paddles were transported to the laboratory, where the presence of eggs was verified using a stereomicroscope. Egg-containing paddles were subjected to immersion in trays containing chlorine-free distilled water and maintained for three days at room temperature to induce hatching. After this period, the paddles were removed and allowed to dry naturally for three days, after which they were subjected to a new immersion cycle, following the protocol established by Alencar et al. (2016) [17]. Hatched larvae were transferred to B.O.D. incubators (Eletrolab, São Paulo, Brazil) under controlled temperature conditions (25 ± 1 °C) and photoperiod (12 h of light and 12 h of darkness).

Specimens were reared to the adult stage, and species identification was performed exclusively on emerged adults. Diagnostic characters were examined under a stereomicroscope (ZEISS Stemi SV61, Jena, Germany), with particular emphasis on male genitalia structures when available. Taxonomic determination followed standard dichotomous keys and species descriptions proposed by Lane (1953) [18], Consoli and Lourenço-de-Oliveira (1994) [19], and Forattini (2002) [20]. All specimens were cataloged and are awaiting voucher number assignment by the Entomological Collection of the Oswaldo Cruz Institute (Fiocruz).

### 2.6. Data Analysis

The Kruskal–Wallis test was used to compare abundance among months, considering only the years from 2022 to 2024, which contained complete 12-month series, whereas the years 2021 and 2025 were excluded from these comparative analyses because they contained partial periods. Data from 2021 and 2025 were included in descriptive and illustrative analyses, thereby enabling visualization of temporal continuity and species fluctuations across all available months, as well as contributing to species-specific temporal trend analyses.

The dataset consisted of 10 ovitraps sampled monthly over 47 months, resulting in 470 trap–month sampling units. For statistical purposes, each trap–month combination was treated as a sampling unit, reflecting the repeated-measures design across spatially replicated traps over time. However, depending on the analytical objective, data were also aggregated at different levels: for temporal comparisons among months, total abundance per month (summing all ovitraps) was used as the response variable, whereas for spatial comparisons among ovitraps, abundance was aggregated across the entire study period for each trap.

The occurrence preferences of vector species in specific months were evaluated using correspondence analysis, in which proximity between a month and a species indicates greater relative abundance during that month; nearby months present similar species composition, and nearby species tend to occur during the same months.

The cumulative percentage of egg hatching was calculated across successive immersions in order to evaluate egg hatchability rates of the studied species. Traps were compared with one another regarding abundance and relative frequency of vector species to assess whether certain species predominated in different traps. In addition, sex ratio calculations were performed for each species. For trap-level comparisons, abundance was aggregated across the entire study period for each ovitrap, allowing evaluation of spatial heterogeneity in species distribution. Statistical analyses were performed using PAST software version 4.05 and R software version 4.3.2.

## 3. Results

**Figure 2:**
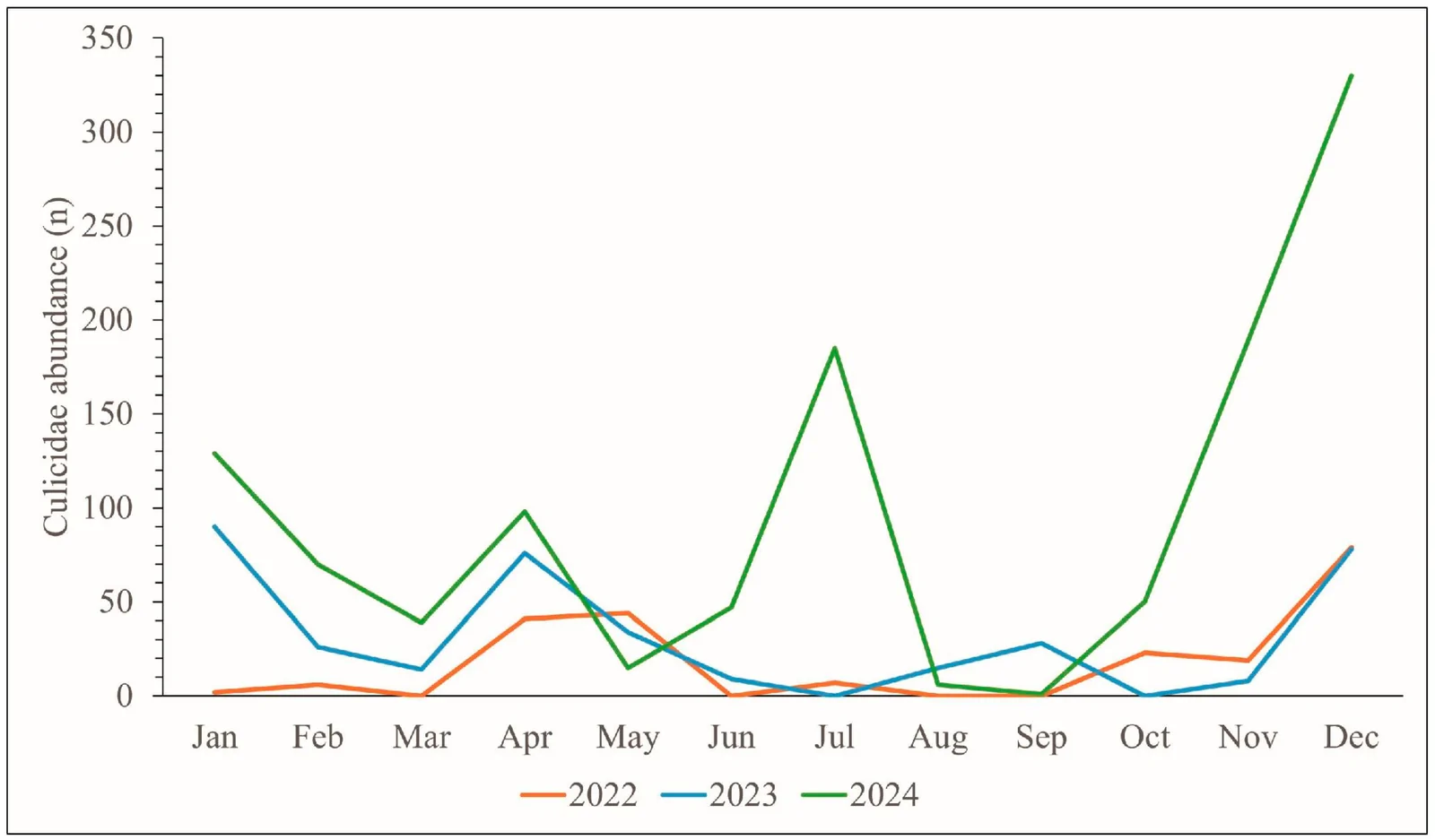
Seasonal trend of medically important mosquito species by month at Sítio Terra Boa, in the municipality of Silva Jardim, state of Rio de Janeiro, Brazil, comparing the collection years 2022, 2023, and 2024.

The population of medically important mosquitoes showed higher abundance in 2024 compared to previous years. Pairwise comparisons following the Kruskal–Wallis test indicated a significant difference only between 2022 (n = 221) and 2024 (n = 1159) (*p* = 0.00261), whereas no significant differences were detected between 2022 and 2023 (*p* = 0.2248) or between 2023 and 2024 (*p* = 0.07243). Considering all years combined, the highest population peaks predominantly occurred during warmer periods, especially in December (n = 487), November (n = 216), January (n = 221), April (n = 215), and July (n = 192). An exception was observed in 2022, when January presented low abundance (n = 2). In contrast, the months with the lowest abundances were those with milder temperatures: August (n = 21), September (n = 29), June (n = 56), and March (n = 53) (Figure 2).

### 3.1. Temporal Trend Analysis of Species Capture

**Figure 3:**
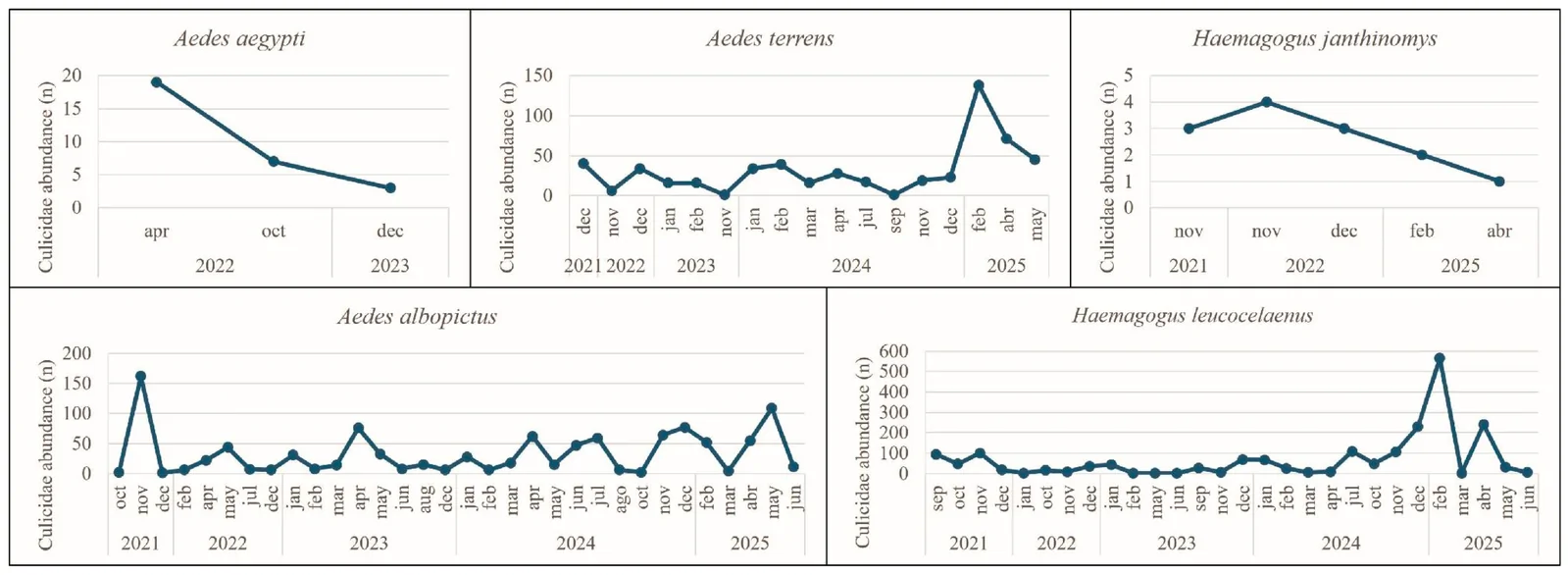
Seasonal trend of medically important mosquito species (*Hg. leucocelaenus*, *Hg. janthinomys*, *Ae. albopictus*, *Ae. terrens*, and *Ae. aegypti*) by year at Sítio Terra Boa, municipality of Silva Jardim, state of Rio de Janeiro, Brazil, from August 2021 to June 2025.

*Haemagogus leucocelaenus* presented the highest abundance values throughout the collection period, with the peak abundance observed in February 2025 (n = 565). Higher abundance values were observed from 2024 onward compared with previous years (2022–2023). *Aedes albopictus* showed abundance peaks in November 2021 (n = 162) and May 2025 (n = 109), whereas *Aedes terrens* exhibited its highest abundance in February 2025, being similar to the pattern observed for *Hg. leucocelaenus*. In contrast, *Hg. janthinomys* maintained low abundance throughout the collection period, with slight increases in November 2021 and 2022 (n = 3 and 4, respectively) and December 2022 (n = 3). *Aedes aegypti* showed low abundance overall, with small increases in April 2022 (n = 19), October of the same year (n = 7), and December 2023 (n = 3) (Figure 3). Overall comparison among species confirmed significant differences in abundance (Kruskal–Wallis χ^2^ = 10.53, df = 4, *p* = 0.032), with post hoc analysis indicating a significant difference between *Hg. janthinomys* and *Hg. leucocelaenus* (Dunn’s test, adjusted *p* = 0.0229), while no other pairwise comparisons remained significant after Bonferroni correction.

### 3.2. Correspondence Analysis

**Figure 4:**
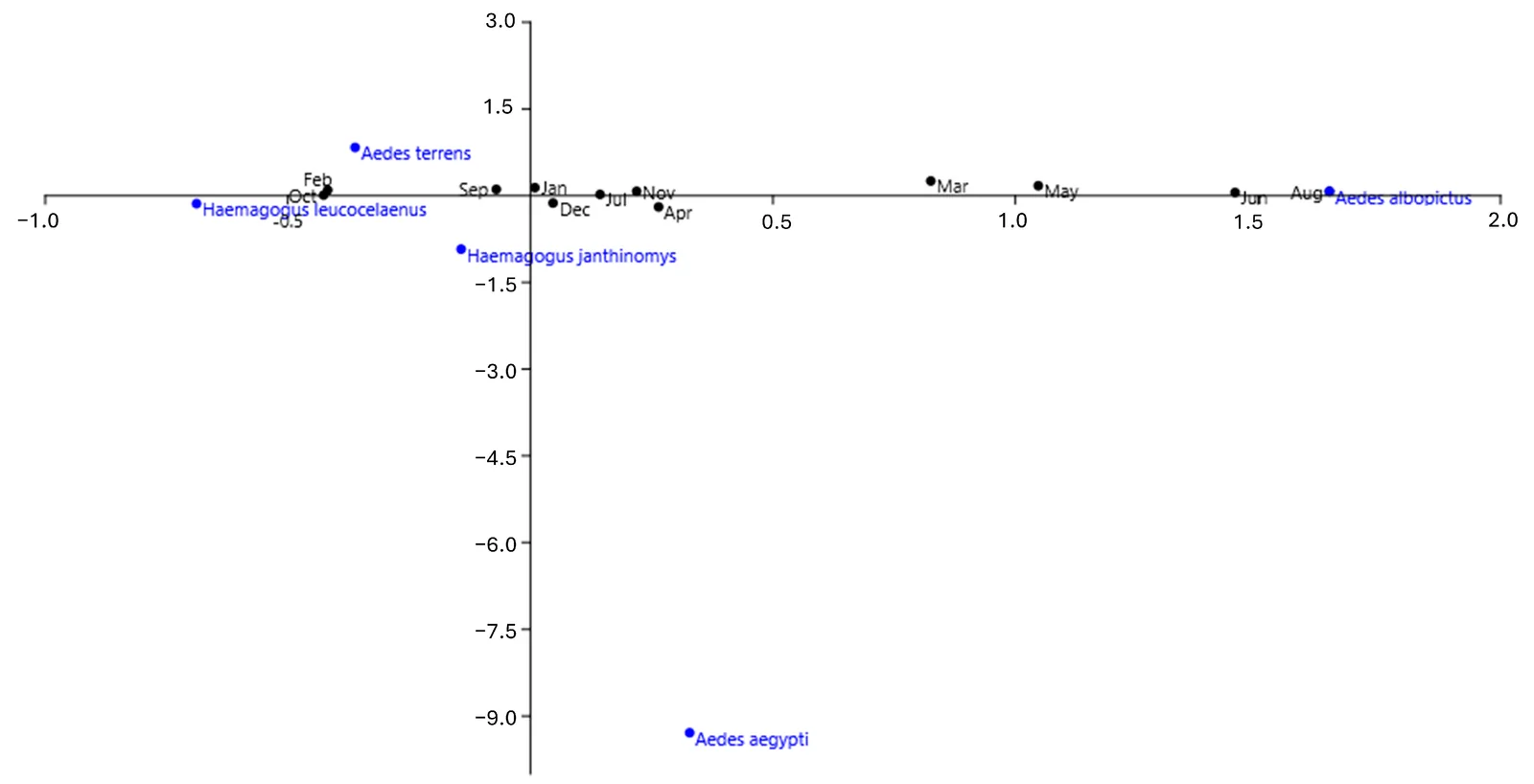
Correspondence analysis between medically important mosquito species (*Hg. leucocelaenus*, *Hg. janthinomys*, *Ae. albopictus*, *Ae. terrens*, and *Ae. aegypti*) and collection months from August 2021 to June 2025 at Sítio Terra Boa, municipality of Silva Jardim, state of Rio de Janeiro, Brazil. Axis 1 explains 91.09% of the variation. Axis 2 explains 8.61%, accounting together for 99.70% of the total inertia.

*Haemagogus leucocelaenus* exhibited the highest relative abundance in October (n = 595), December (n = 577), February (n = 535), November (n = 131), and January (n = 112). Similarly, *Aedes terrens* also demonstrated a strong association with these months: February and October (n = 194 for both), December (n = 158), November (n = 55), September (n = 51), and January (n = 50). *Haemagogus janthinomys* was also recorded during November (n = 3), October and February (n = 2 for both), and December (n = 1), being absent during the remaining study months. The *Aedes albopictus* and *Ae. aegypti* species displayed the most distinct patterns. *Aedes albopictus* showed a differentiated pattern, with greater abundance in December (n = 263), May (n = 201), April (n = 174), November (n = 80), July and October (n = 72 for both), and June (n = 71). This species also exhibited higher abundance than the other vector species during months such as March (n = 35) and August (n = 23), with the latter month showing exclusive occurrence of this species and total absence of all others. *Aedes aegypti* was only recorded in December (n = 22), April (n = 19), and October (n = 7) (Figure 2). The months of February and October presented similar species composition, characterized by high abundance and the presence of most vector species evaluated in the present study (Figure 4).

### 3.3. Immersions

**Figure 5:**
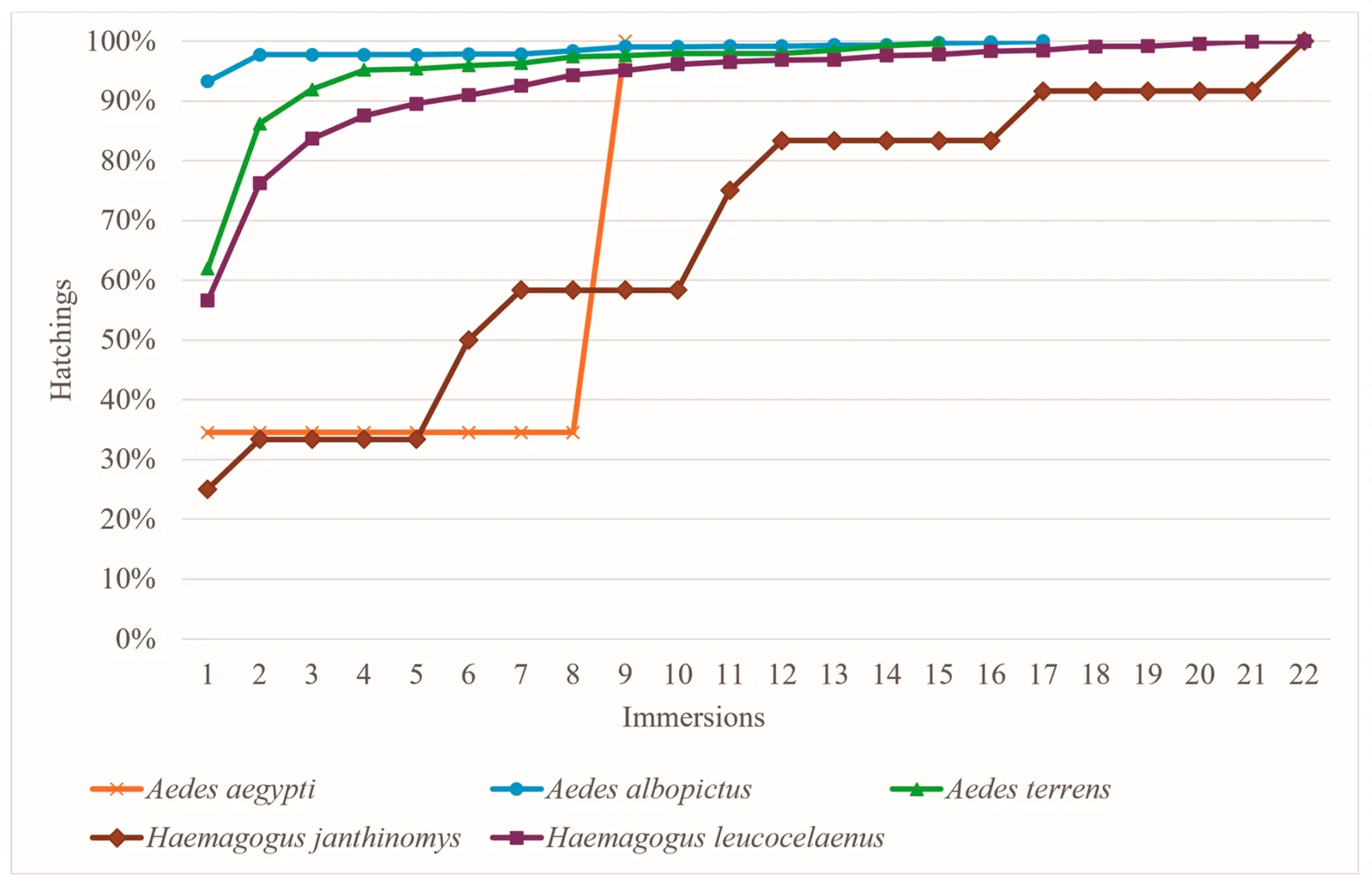
Percentage of cumulative hatching throughout successive immersions of eggs from medically important mosquito species (*Hg. leucocelaenus*, *Hg. janthinomys*, *Ae. albopictus*, *Ae. terrens*, and *Ae. aegypti*) collected at Sítio Terra Boa, municipality of Silva Jardim, state of Rio de Janeiro, Brazil, from August 2021 to June 2025.

A total of 29 eggs of *Ae. aegypti*, 1056 eggs of *Ae. albopictus*, 544 eggs of *Ae. tereens*, 12 eggs of *Hg. janthinomys* and 1904 eggs of *Hg. leucocelaenus* were analyzed in the hatching experiments. *Aedes aegypti* eggs reached complete hatching by the 9th immersion, with two major hatching events: an initial event during the 1st immersion and another during the 9th immersion. *Aedes albopictus* eggs exhibited high hatching rates already during the first immersion, with slight increases during subsequent immersions, reaching 100% hatching by the 17th immersion. Similarly, *Aedes terrens* eggs showed a progressive increase in hatching throughout the immersions, reaching complete hatching by the 15th immersion. In contrast, species of the *Haemagogus* genus required a greater number of immersions to achieve complete egg hatching. Both *Hg. janthinomys* and *Hg. leucocelaenus* exhibited hatching events distributed throughout all immersions, reaching 100% hatching only by the 22nd immersion (Figure 5).

It is noteworthy to observe the hatching behavior pattern of *Hg. leucocelaenus*, *Ae. albopictus*, and *Ae. terrens*. These species displayed a gradual distribution of hatching events throughout the immersions. However, *Ae. albopictus* and *Ae. terrens* exhibited higher hatching rates during the initial immersions, reaching complete hatching earlier (17th and 15th immersions, respectively) when compared with *Hg. leucocelaenus*, which exhibited a more prolonged pattern (22nd immersion) (Figure 5).

### 3.4. Comparison of Abundance Among Traps

*Haemagogus leucocelaenus* was the most abundant species in most traps, with relative frequencies ranging from 26.37% (Trap 5) to 73.53% (Trap 1), indicating its predominance at the collection site. *Aedes albopictus* exhibited moderate frequencies, with values ranging from 7.69% (Trap 3) to 73.63% (Trap 5), being the most abundant species in traps 4 to 7. *Aedes terrens* displayed a more irregular distribution, with frequencies ranging from 0% (Trap 5) to 33.47% (Trap 7), particularly standing out in traps 2 (26.22%) and 7 (33.47%), indicating a heterogeneous spatial distribution.

*Aedes aegypti* was infrequently recorded, only being detected in traps 8 (3.6%) and 10 (1.95%), suggesting low occurrence or restricted preferences for specific collection sites. Finally, *Hg. janthinomys* was detected at low frequencies exclusively in traps 9 (1.5%) and 10 (5.84%), indicating a rare or sporadic occurrence within the studied environment.

**Figure 6:**
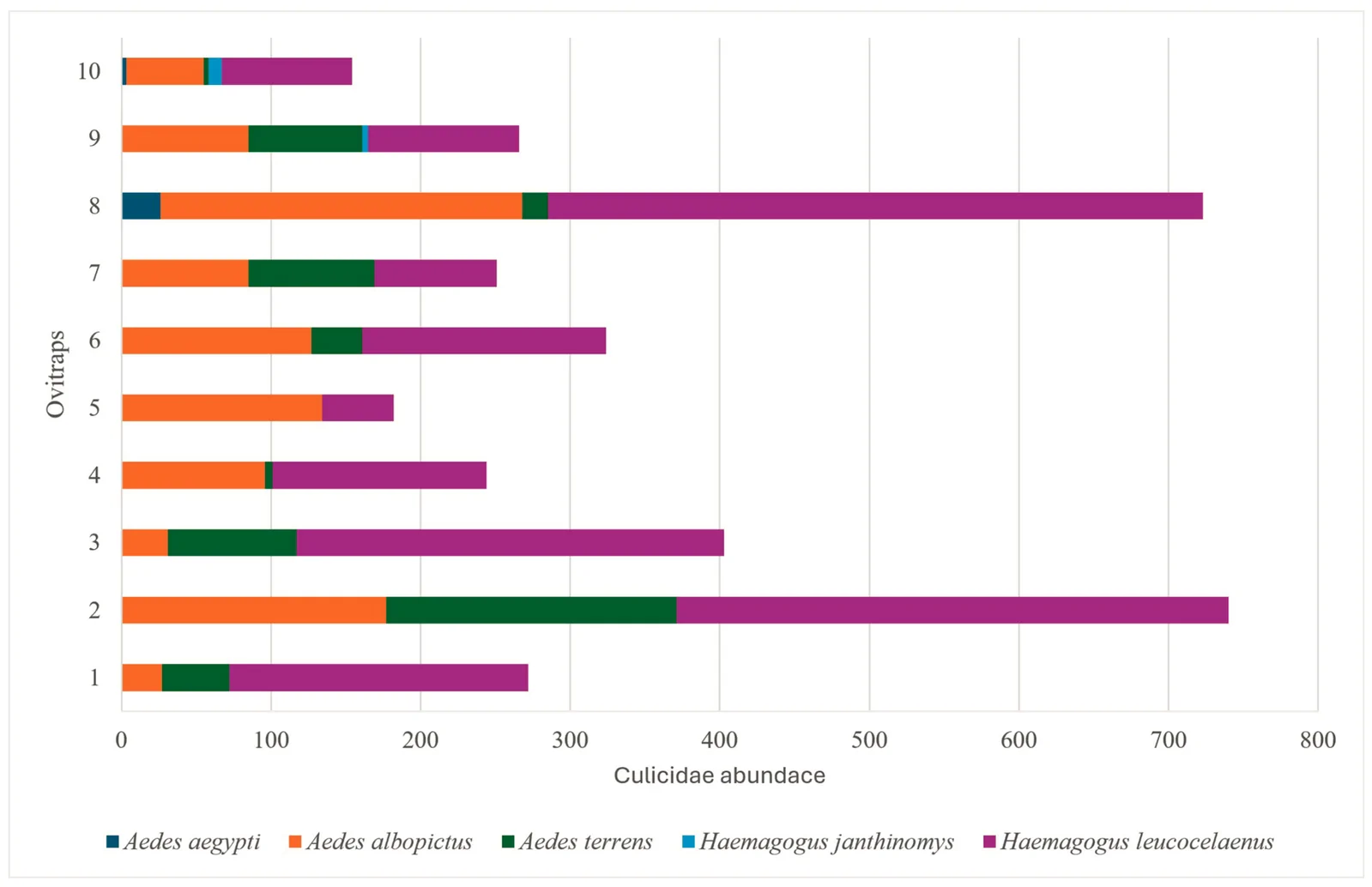
Abundance of medically important mosquito species (*Hg. leucocelaenus*, *Hg. janthinomys*, *Ae. albopictus*, *Ae. terrens*, and *Ae. aegypti*) by trap collected at Sítio Terra Boa, municipality of Silva Jardim, state of Rio de Janeiro, Brazil, from August 2021 to June 2025.

Overall, a predominance of *Hg. leucocelaenus* was observed among the traps, whereas *Ae. albopictus* and *Ae. terrens* showed relevant frequencies at specific sampling points. In contrast, *Ae. aegypti* and *Hg. janthinomys* exhibited restricted and sporadic occurrence (Figure 6).

### 3.5. Sex Ratio by Species

Analysis of the sex ratio (M/F) revealed variations among the sampled Culicidae species. *Aedes aegypti* presented a sex ratio of 0.71, indicating female predominance. *Aedes albopictus* exhibited a sex ratio slightly above 1 (1.04), suggesting a balanced proportion with a slight predominance of males. *Aedes terrens* presented a sex ratio equal to 1, indicating an equal proportion between sexes. The sex ratio for *Haemagogus janthinomys* was 1.17, indicating a greater occurrence of males, whereas *Haemagogus leucocelaenus* showed the lowest sex ratio (0.68), with predominance of females (Table 1).

## 4. Discussion

The study of mosquito communities in natural environments under anthropogenic influence, conducted in an Atlantic Forest fragment in Rio de Janeiro, enabled identifying five epidemiologically relevant species collected through ovitraps: *Hg. leucocelaenus*, *Ae. albopictus*, *Ae. terrens*, *Ae. aegypti*, and *Hg. janthinomys*. Considering all traps together, a predominance of *Hg. leucocelaenus* was observed in virtually all ovitraps, reinforcing the role of this species as the main component of the Culicidae fauna in the studied area.

*Haemagogus janthinomys* is recognized as the primary vector of the sylvatic yellow fever virus in the Americas and has also been associated with the transmission of other arboviruses, such as Mayaro and Ilhéus viruses [21,22]. In turn, *Hg. leucocelaenus* is considered a primary vector of yellow fever virus in southeastern Brazil [19,23] and has also been implicated in the circulation of viruses such as Ilhéus, Maguari, and Tucunduba [24]. *Ae. aegypti* stands out among the aedines as the principal vector of dengue, Zika, Chikungunya, and urban yellow fever viruses in Brazil, whereas *Ae. albopictus* is considered a potential vector of dengue, yellow fever, and Chikungunya viruses [25,26,27]. Additionally, the epidemiological importance of *Ae. terrens* has been suggested based on high infection and dissemination rates of Chikungunya virus under experimental conditions [28].

In this context, the present findings contribute to a better understanding of the ecology of medically important mosquito species in modified natural environments, particularly those subjected to açaí (*Euterpe oleracea*) and peach palm (*Bactris gasipaes*) cultivation within an Atlantic Forest remnant. The higher abundance of *Hg. leucocelaenus* in the ovitraps suggests that this species may persist and reproduce in agroforestry environments. However, comparative studies including different landscape types are needed to evaluate its ecological adaptation. The coexistence of species from the *Haemagogus* and *Aedes* genera within the same forest fragment under anthropogenic interference highlights the ecological complexity of these interface environments and reinforces the importance of continuous entomological surveillance. These findings provide valuable information on the composition and seasonal dynamics of medically important mosquito species in agroforestry systems and provide a basis for future comparative studies in other agroforestry systems and landscape contexts.

Mosquito populations exhibited a well-defined seasonal pattern, with greater abundance during warmer periods (December to April), especially in February and October, and lower population densities during colder months (June to September). Previous studies have shown that higher temperatures, associated with increased humidity and rainfall, may favor breeding site availability and promote larval development and survival, resulting in increased Culicidae population density [29]. The seasonal pattern observed in the present study is consistent with these findings; however, climatic variables were not directly measured.

*Haemagogus leucocelaenus* followed this pattern. It is known that breeding sites used by species of the *Haemagogus* genus are strongly influenced by fluctuations in water levels and may undergo desiccation during drier periods. Eggs of these species are deposited on moist plant substrates near the water surface and require successive flooding events for hatching, allowing a single oviposition event to result in adult emergence over different periods [30]. In this context, warm and rainy periods provide greater frequency of egg immersion events, where the combination of high temperatures and increased precipitation (typical of the rainy season) contributes to higher hatching rates and accelerated larval development [31].

Similarly, *Ae. terrens* followed the same pattern as *Hg. leucocelaenus*, suggesting an analogous ecological response to environmental conditions. In contrast, *Ae. albopictus* showed broader distribution throughout the year, including colder months, corroborating its recognized ecological plasticity and ability to occupy both sylvatic and peri-urban environments [24].

This temporal differentiation among species reveals important implications for entomological surveillance. Whereas *Hg. leucocelaenus* and *Ae. terrens* concentrate their greatest seasonal activity during warm and rainy months, *Ae. albopictus* maintains continuous activity throughout the year, remaining present even during periods which are less favorable to other species. If future studies demonstrate that these periods are associated with increased arbovirus transmission risk, entomological surveillance could be intensified during this seasonal window. However, specific control recommendations require additional studies directly assessing arbovirus infection and adult mosquito abundance. Thus, vector management approaches in the studied area should not only consider species composition, but also the distinct seasonal patterns of each species, adjusting monitoring and control strategies according to the observed temporal dynamics. This ecological consistency reinforces the need for surveillance programs integrating climatic and biological variables to support surveillance and ecological monitoring at the interface between natural environments and areas cultivated with açaí and peach palm.

In: the present study, most *Ae. albopictus* eggs hatched during the first immersion in water, corroborating patterns previously described in Atlantic Forest remnants in Rio de Janeiro and Minas Gerais [31,32,33]. Most *Ae. terrens* egg hatching occurred between the 1st and 4th immersions, although cumulative effects were observed up to the 15th immersion. Similar patterns were described by Alencar et al. (2014) [32] and Silva et al. (2023) [34], who reported greater hatching concentration during the first immersions, with cumulative effects extending up to the 13th and 12th immersions, respectively, suggesting consistency in this species’ response pattern to successive flooding events across different Atlantic Forest areas. Complementarily, Galindo et al. (1955) [35] observed that most hatching occurred after the first flooding event in field-collected eggs from Panama.

The requirement for multiple immersions to achieve complete egg hatching for species of the *Haemagogus* genus has also been widely reported in the literature [34,36]. In this context, Alencar et al. (2008) [37] demonstrated that *Hg. janthinomys* eggs hatch in batches following successive flooding events, remaining viable even after multiple immersions. This adaptive behavior allows eggs from a single oviposition event to respond in a staggered manner, with hatching triggered only after sufficiently intense rainfall events to promote complete submersion.

From an ecological perspective, this staggered hatching pattern provides significant advantages for Culicidae populations. In environments subjected to seasonal rainfall patterns, such as Atlantic Forest fragments under anthropogenic influence, the gradual emergence of adults from a single oviposition event reduces the probability of local population extinction during dry periods or adverse climatic events. This mechanism may contribute to the persistence of *Hg. leucocelaenus* and *Hg. janthinomys* populations even after prolonged periods without precipitation, since quiescent eggs remain viable in the environment until favorable conditions for hatching occur. Likewise, the early hatching strategy of *Ae. albopictus* reinforces its colonization potential and rapid occupation of temporary breeding sites, whereas the more gradual response of *Ae. terrens* indicates an intermediate strategy, possibly associated with greater tolerance to hydrological variation. Such interspecific differences may have important implications for the population dynamics of mosquito species in environments subjected to hydrological variation. Understanding these patterns may contribute to future ecological studies and support the planning of entomological surveillance in similar agroforestry systems.

From an ecological perspective, the greater proportion of females observed in certain species is noteworthy, since only females perform hematophagy and actively participate in pathogen transmission [19]. Thus, the predominance of *Hg. leucocelaenus* associated with the higher frequency of females is epidemiologically relevant in environments under anthropogenic influence. Although *Hg. janthinomys* showed male predominance, which in isolation suggests lower immediate vector risk, its mere presence in the environment cannot be disregarded, since even populations with male-biased sex ratios may contribute to species maintenance and to the eventual emergence of infected females under favorable conditions. Further studies evaluating adult female abundance and host-feeding activity are needed to better understand the ecological significance of these patterns.

Agroforestry systems within the Atlantic Forest may sustain Culicidae communities of high epidemiological relevance, particularly due to the dominance of *Hg. leucocelaenus*. The co-occurrence of species with different degrees of ecological plasticity, including *Ae. terrens*, *Ae. albopictus*, and *Ae. aegypti*, indicates that the açaí and peach palm agroforestry system evaluated can function as an ecological interface supporting multiple vector species. These findings reinforce the importance of continuous and ecologically oriented entomological surveillance strategies in areas where environmental changes may influence mosquito community structure.

Finally, our study demonstrated that Atlantic Forest fragments cultivated with açaí (*Euterpe oleracea*) and peach palm (*Bactris gasipaes*) harbor a diversity of mosquito vector species, with clear dominance of *Hg. leucocelaenus* and seasonality, marked by warmer and rainier periods. *Aedes albopictus* stood out due to its broad temporal distribution and high ecological plasticity, whereas *Ae. terrens* and *Hg. janthinomys* exhibited more restricted patterns. Additionally, hatching experiments revealed some distinct flooding-response strategies: *Ae. albopictus* showed massive hatching during the first immersion, *Ae. terrens* displayed a gradual response, and *Haemagogus* species exhibited staggered hatching across multiple events, conferring population resilience under rainfall variability. The male-biased sex ratio observed in *Hg. janthinomys* contrasted with the predominance of females in *Hg. leucocelaenus* and *Ae. aegypti*, which, together with the abundance and dominance of the former species, positions *Hg. leucocelaenus* as the principal potential vector in the studied area.

## 5. Conclusions

Based on the findings presented, it can be concluded that the agroforestry system evaluated in this study, comprising açaí and peach palm cultivation within an Atlantic Forest remnant, supports a diverse community of Culicidae species of epidemiological relevance. The dominance of *Hg. leucocelaenus*, the predominance of females of this species, and its staggered hatching strategy indicate that this specific agroforestry system provides favorable conditions for the persistence of vector populations. However, the public health implications of these findings should be interpreted with caution. The results support the inclusion of this specific type of agroforestry system in regular entomological surveillance programs in southeastern Brazil. Further comparative studies across different agroforestry systems, monocultures, and forest remnants are necessary to determine whether these patterns are generalizable to other anthropogenically modified environments. Integrated environmental management measures may be considered for this and similar systems, but their effectiveness should be evaluated through targeted studies.

## Figures and Tables

**Table 1:** Sex ratio by species collected at Sítio Terra Boa, municipality of Silva Jardim, state of Rio de Janeiro, Brazil, from August 2021 to June 2025.

Species	F	M	Male/Female Ratio
*Aedes (Stegomyia) aegypti* (Linnaeus, 1762)	17	12	0.71
*Ae. (Stegomyia) albopictus* (Skuse, 1894)	517	539	1.04
*Ae. (Protomacleaya) terrens* (Walker, 1856)	272	272	1.00
*Haemagogus (Haemagogus) janthinomys* Dyar, 1921	6	7	1.17
*Hg. (Conopostegus) leucocelaenus* (Dyar & Shannon, 1924)	1142	775	0.68

## Data Availability

The original contributions presented in this study are included in the article. Further inquiries can be directed to the corresponding author.
